# ‘Accident and emergency’? Exploring the reasons for increased privatisation in England's NHS

**DOI:** 10.1016/j.healthpol.2023.104941

**Published:** 2023-12

**Authors:** Benjamin Goodair

**Affiliations:** Department of Social Policy and Intervention, University of Oxford, Barnett House, 32-37 Wellington Square, Oxford OX1 2ER, United Kingdom

**Keywords:** NHS, Privatisation, Outsourcing, Commissioning, Health policy, Quality, Contracting-out

## Abstract

England's NHS is experiencing rising privatisation as services are increasingly being delivered by private healthcare providers. This has led to concerns about the supposed benefit of this process on healthcare quality but the reasons for the increase – and whether processes prioritise quality – are not well understood. In-depth semi-structured interviews with 20 people involved in the commissioning process, sampled from 3 commissioning sites (regional health boards) are thematically analysed. Four key themes of reasons for outsourcing were identified: unmet need; the “choice agenda”; appetite for change amongst key individuals working at the commissioning body; and the impact of financial pressures. The study concludes that the experience of commissioners navigating the provision of healthcare with worsening social determinants of health and financial austerity means that decisions to use private providers based on anticipated quality are sometimes but not always possible - sometimes they constitute ‘accidents’, sometimes ‘emergencies’.

## Highlights

• England's NHS is being privatised as services are outsourced to private providers.
• But why privatisation happens, and whether it prioritises ‘quality’ is unknown.
• This study interviews healthcare commissioners and asks why services are outsourced.
• Commissioners are responding to unmet need, national guidelines, financial pressures and politics.
• Some instances of privatisation fail to prioritise quality in the outsourcing process.

## 1 Introduction

England's National Health Service has observed a 20-year period of privatisation through increases in outsourcing of clinical healthcare provision to the private sector. Services are still funded by the NHS, paid largely out of general taxation and, mostly, free at the point of use – but delivered by a private company. Consecutive UK Governments have introduced national regulation to significantly empower private companies to deliver treatments instead of NHS providers. In theory, the NHS is designed so that new private providers enter the ‘market’ once they are actively selected as the best quality alternative to provide healthcare – either through patients choosing their preferred provider or through the commissioning of services by regional health boards (named Clinical Commissioning Groups, CCGs, at the time of research). This kind of state-funded and mix of public and privately-provided service model is common in many country-contexts as instances of health and care services have frequently been ‘outsourced’ to the private sector [1,2].

The implementation of this form of ‘privatisation via outsourcing’ is founded on, and justified by, a theory that it *improves* their quality of service [3]. A common underlying assumption is that the provision of services are gatekept by state-run organisations that actively select service providers on perceived quality [4,5]. But whether this happens in practice is not well known. This paper provides the empirical basis for testing whether processes of privatisation in England actively select on quality of care.

### 1.1 The privatisation of the NHS and the quality of care

Over the last 40 years, since the first efforts to introduce of the internal market to the NHS, UK governments have made consistent claims that a process of privatisation will improve the quality of services delivered by the NHS in England. Indeed, David Cameron, former Prime Minister, noted in 2011 that the “*NHS has always benefited from a mixed economy of providers”* and that creating space for new private providers will only create *“more choice and competition”* and thereby *“raises standards and delivers values for money.”* [6]. Cameron was not alone. Such arguments were commonly made by Tony Blair during the early 2000s and Labour's 2001 manifesto read “*Where the quality is not improving quickly enough, alternative providers should be brought in. Where private-sector providers can support public endeavour, we should use them*.” [7].

**Fig. 1:**
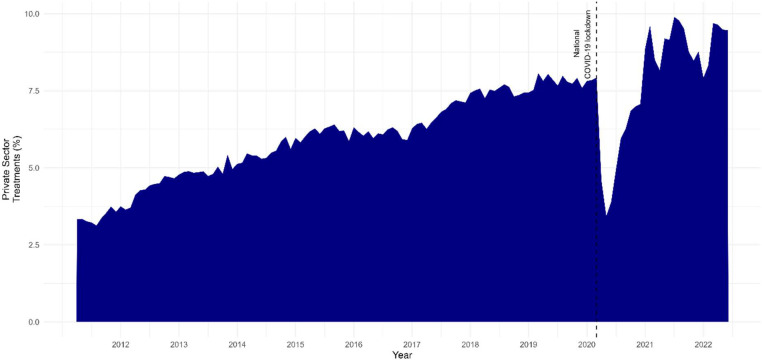
Scale of increase in private provision of NHS services 2011–22. Data source: RTT waiting time data (NHS England, 2022). Replication materials available at https://github.com/BenGoodair/NHS_privatisation_treatments

During the last decade the increases in private sector provision have been sizeable, Fig. 1 shows that increases in the number of private treatments have increased from 3% to 9% since 2011 when data was first produced on treatment provision. Figure A6 in the Appendix shows that the amount of outsourcing varies widely by the type of treatment – but also that most treatments have seen increases over this period. Thanks to the development of transparency data in public bodies, and the dedicated work to harmonise these resources, we are now also able to trace public expenditure and link it to the individual suppliers [8], [9], [10]. This has enabled researchers to detail how expenditure on private sector suppliers has consistently increased over the last decade, resulting in billions more pounds spent on private sector providers [10,11].

However, there is no evidence that the trend of increased privatisation has corresponded with better quality care. One body of evidence has analysed whether health outcomes for patients differ for those carried out in private hospitals compared to NHS hospitals. It tends to find little difference in health outcomes or patient satisfaction between the sectors but always stresses that direct comparisons are hard to make given the different characteristics and health needs of patients in the different sectors [12], [13], [14].

When studied at an aggregate level, findings suggest that increases in outsourcing are associated with worse health outcomes in England [11]. An association between increased for-profit provision and rises in mortality rates was also observed in Italy during a period of privatising reforms [15]. The benefit of these studies is that they are not prone to the issue of comparing providers which are performing different duties. However, aggregate level studies cannot easily identify why privatisation is associated with higher mortality rates.

This debate can be contextualised within a much wider literature about the impacts of ownership on the quality of services. In adult and children's social care services in England, a consistent trend indicates higher quality in publicly owned provision [16], [17], [18]. Internationally a ‘for-profit gap’ in quality of healthcare is frequently observed in the USA [15,19,20]. For non-clinical services in the NHS, although the processes and conditions might be very different to clinical services, negative outcomes have been associated with outsourced cleaning and management [21,22].

There is, however, a very considerable gap in our understanding of the *process* of privatisation within healthcare services. In other words, why is it the case that NHS commissioners are increasingly using private providers? Answering this question is critical if we want to understand why privatisation might be associated with some of the health harms outlined above. If, for example, NHS privatisation is not occurring in pursuit of higher quality then this might help us understand why such shifts have been associated with poorer outcomes in some instances.

### 1.2 Towards a quality-centred typology of outsourcing

In this paper I will introduce and utilise a novel framework to help think about the process of outsourcing – and how it may correspond with quality of care: controlled vs uncontrolled outsourcing and quality-prioritising vs quality-neglecting outsourcing.

Previous typologies have been used to identify varieties of privatisations or types of markets. For instance, one literature creates a typology of privatisation along three dimensions: funding, decision-making, and provision [23], [24], [25]. This work often describes the privatisation of England's NHS as ‘contracting-out’ or outsourcing – because the funding and decision-making is in the public realm but the provision is being transferred to private providers [26,27]. Similarly, Gingrich creates a typology for the market conditions in which public services are provided– along the dimensions of who has control over production (the state, companies, or individuals) and whether responsibility for access to services are decided individually or collectively [28]. Here, the NHS in England might be considered a ‘managed market’ where collective decisions are made about who can access services and the state controls the types of services provided. However, one of the arguments of this paper will be that processes of marketisation and outsourcing are highly varied even within a country's health service.

These existing ways of categorising privatisation are, to some extent, neutral to the idea of quality of those services – something this paper attempts to add with an alternative framework building on the concept of ‘outsourcing’.

Key to the processes of privatisation in England's NHS are four ‘sites of power’ and decision-making: 1) central government; 2) commissioners; 3) providers (acting as sub-contracting bodies) and 4) patients. If this process is to work as theorised, an important question exists for each site of power – ‘*whether the decision to increase the use of private providers has at its core the intention to improve healthcare quality*’. If this condition is not met at any one site, then privatisation could have either a) occurred passively and unintentionally – as ‘uncontrolled outsourcing, or b) because of a failure to incentivise or empower the key actors to prioritise healthcare quality – as ’quality-neglecting outsourcing’.

For example, taking the site of commissioners, uncontrolled outsourcing could happen through commissioners having no power to actively determine the selection of providers; whereas quality-neglecting outsourcing could occur through commissioners being incentivised to procure a service based on priorities which come at the expense of quality of care. The utility of this heuristic is that it lets us identify the processes of privatisation which do not prioritise quality – either by accident or on purpose.

The ’control’ dimension of this framework is not dissimilar to the ‘decision-making dimension’ used in other conceptualisations [23]. It differs, however, because the ‘decision-making’ dimension is interested in whether power lies with a private individual or a public body – but for the NHS it may be the case that neither is true and that the market has been captured by private companies. By focusing on commissioner control, we can identify moments where neither service-users nor public bodies have decision-making power about who delivers healthcare.

The ’quality’ dimension of this framework attempts to identify when outsourcing is justified by alternative priorities to improving the quality of services. This is important because it might help explain when privatisation may lead to worse outcomes. It is important to note that commissioners are expected to make their decisions based on a range of factors including value, access to services, social value, and sustainability [29]. Consequently, a commissioner might prioritise another of those factors and be acting perfectly within the official guidance.

To better understand which scenario best explains recent privatisation developments in the NHS, this paper aims to provide an empirically informed discussion addressing whether privatisation is an active attempt to improve the healthcare in the English NHS; or an unintended phenomenon; or process which does not centre quality of care.

This paper operationalises the perspectives and experiences of employees at one key site of power – healthcare commissioners - asking them why they perceive private providers to be used in the NHS – and identifies moments of uncontrolled and quality-neglecting outsourcing – or ‘accidents’ and ‘emergencies’.

## 2 Materials and methods

### 2.1 Participant selection and data collection

To answer these questions, I followed a qualitative research design, conducting in-depth, semi-structured interviews with individual healthcare commissioners within three commissioning bodies. Three commissioning bodies (CCGs) were selected purposively on the sole criterion of levels of private provision, with two CCGs which had high levels of private sector provision and one CCG with particularly low levels of private sector provision based upon analyses of commissioner expenditure data [10]. Specifically, invites were sent to the most and least outsourcing CCGs, many did not respond to requests and therefore the selected CCGs are ‘amongst the most and least outsourcing CCGs’. The range intended to return a variety of experiences with the private sector to identify the ‘extreme cases‘ on the independent variable as proposed by Seawright [30]. After selecting key sites, a preliminary discussion was held at each site with a gatekeeper in a leadership position who subsequently helped recruit the individuals for interviews. I requested participants from each site who worked in different settings to attain insights from a diverse range of the commissioning process. Nine participants had leadership job roles, and nine participants were also selected from a low outsourcing commissioner setting. Further participant details are available in the appendix (Tables A1 and A2).

I conducted interviews from June to September 2022. The recruited participants worked at 3 CCG sites (or previously worked at those sites before reforms removed them in the summer of 2022) and interviews lasted 45–70 min. The semi-structured process loosely followed an interview guide. Most of the time was spent discussing responses to two open questions of a) perceived reasons for the NHS using the private sector to deliver health treatments and b) what they think explain variation in levels of private sector outsourcing amongst different CCGs. Some topics were included as prompts if not raised by the interviewees themselves. These prompts included questions such as whether private providers offered better or cheaper services than NHS providers; the role of commissioner finances; and the impact of individuals in leadership positions at the commissioning site. Interviews were conducted until the data was saturated after 20 interviews [31].

### 2.2 Analysis

I conducted thematic analysis on the interview transcripts [32]. Initial codes were created underneath the two broad categories, which were defined a priori, of ‘why use private providers’ and ‘why would private usage vary between commissioners’? Organising themes were then inductively produced from the basic codes in the data. The results are presented with a section for each organising theme and a table linking basic codes to organising themes, with an example quotation. This can be found in Table 1 and in the appendix along with a discussion of reflexivity, researcher positionality and ethics (Table A3, A4, A5).

**Table 1:** Varieties of privatisation in England's NHS.

Organising Theme	Basic Theme	‘Uncontrolled’ or ‘Quality-neglecting’ privatisation at the commissioner level?
Failure of NHS providers in meeting demand: the public sector underfunded and private sector as ‘a release valve’	Longer waiting times increase use of private sector.	‘Quality-neglecting’ – often prioritises quantity of provision, rather than quality. Enforced by limited NHS capacity.
	Varying population demographics would alter need for, and uptake of, private sector.	Could be ‘uncontrolled’ for commissioners but this theme often identified power in the hands of the patients.
	Quality metrics and user feedback may lead to use of competitive procurement process.	Neither – prioritises quality and locates power with the commissioners.
	Absence of NHS provision, often for siloed services.	‘Uncontrolled’ – power lies with ‘the market’ of provision.
	Workforce availability limited NHS expansion.	‘Uncontrolled’ – power lies with central workforce policies.
Private provider locations and the choice agenda	Privatisation driven by locations of private hospitals.	‘Uncontrolled – commissioners do not have the power to control the healthcare market.
	New Labour reforms created the situ of private hospitals through ISTC contracts.	‘Uncontrolled’ at the site of commissioners but the key site of power was central government in this theme.
	Rural commissioners have limited access to private provision.	‘Uncontrolled’ – commissioners do not have the power to control the healthcare market.
	‘The Choice Agenda’ and predatory providers empower private providers.	‘Uncontrolled’ – No power with the commissioner to dictate the provision. It was suggested this could be an ‘Quality-neglecting’ at the patient level through direct-to-consumer advertising.
Commissioning Leadership and Politics	Leader's appetite for alternatives.	Neither – always with the aim of improving quality of care and locates power with the commissioners.
	Outsourcing to challenge NHS provider cultures.	‘Quality-neglecting’ – not clear that the cultures being challenged were primarily about health service quality.
Failure of NHS providers in meeting demand: the public sector underfunded and private sector as ‘a release valve’	Longer waiting times increase use of private sector.	‘Quality-neglecting’ – often prioritises quantity of provision, rather than quality. Enforced by limited NHS capacity.
	Varying population demographics would alter need for, and uptake of, private sector.	Could be ‘uncontrolled’ for commissioners but this theme often identified power in the hands of the patients.
	Quality metrics and user feedback may lead to use of competitive procurement process.	Neither – prioritises quality and locates power with the commissioners.
	Absence of NHS provision, often for siloed services.	‘Uncontrolled – power lies with ‘the market’ of provision.
	Workforce availability limited NHS expansion.	‘Uncontrolled’ – power lies with central workforce policies.
Consequences of Financing and Austerity	Stringent budgets induce outsourcing	’Quality-neglecting’ – prioritises finances.
	Stringent budgets constrain outsourcing	‘Quality-neglecting’ – prioritises finances.
	Prices of comparable services don't generalise by sector of provision	‘Quality-neglecting’ – prioritises finances.

## 3 Results

### 3.1 ‘It depends what's on your plot – build another lane on the M25 and people will drive on it’: Private hospital locations, the choice agenda and predatory providers

A common response to the question of ‘why does private sector usage vary between regions?’ was that it would depend on whether there were private healthcare providers ‘on your patch’. This theme of responses highlighted how subject the decisions of commissioners were to the available ‘market’ of providers – it suggests there are times when commissioners have little power to control outsourcing and might lead to uncontrolled outsourcing. Participant N (CCG setting #3) discusses the procurement of services and explains that:

“I think probably the biggest reason is location. And the number of providers around you. You know, it is a hell of a lot easier [to put a] service out for procurement when you've got a massive population and a lot of different providers.”

The rurality of the CCG was generally addressed as something which led to a dearth of private sector providers and would result in low levels of private sector provision. Participant D (CCG setting #1) said:

“Trying to attract an independent provider. Because we are in such geographical corner is quite challenging. It is relatively unique to [this area] because there's very few major cities that are so far away.”

One of the perceived causes of private provider availability in any geographic area were the centrally agreed NHS contracts for private provision promoted by the mid-2000s New Labour's Government termed *Independent Sector Treatment Centres* which were frequently cited as key in developing the relationships required between commissioner and provider. Participant M (CCG setting #3) described it as “*a national procurement exercise which drew in international capacity and capability*”. If an area had an independent treatment centre contract in 2006, it was perceived they might still have higher levels of outsourcing as a result even 15 years later.

Underpinning many of the discussions on provider location was how ‘the choice agenda’ meant that commissioners were sometimes obliged to fund a variety of providers for patients to choose from. For some commissioners this was discussed in a neutral or positive light and often justified based on it being in the NHS constitution. For example, Participant O (CCG setting #1) said “*you have the right choose”* because of “*the skills you want, or based on waiting times*” and that “*it's written in the constitution… it's fundamental”.* Several participants also reported that they thought patients generally preferred private options when offered for a range of reasons which included the upkeep of facilities and a middle-class ‘aspiration’ for private providers.

However, this framework has also enabled some more problematic practices in the views of the interviewed participants. Commissioners reported that private providers could create a demand for treatments by aggressively advertising the services or they could simply treat more people by exploiting contracts with wider specifications on the services. A similar practice of ‘direct-to-consumer’ advertising for healthcare is common in the USA and is of concern to many suggesting it may work against the goal of improving healthcare quality [33]. At its worst for the participants, the direct-to-consumer advertising in England led to patients being treated who it was perceived did not need a procedure. Commissioners sometimes noted that patients’ best interests were not being prioritised by the private providers delivering cataract surgeries. Participant F (CCG setting #2) said:

“some of these companies do a lot of marketing. So we've had leaflets sent to just about every household, and some of them are linked as well to primary care services to have a direct link through…”

This was explained as being potentially problematic because it impacts the finances of the CCG and also has the potential to put patients at risk of surgery-related issues without being properly advised about those risks.

“Participant F: There are some people though, that will have problems with their vision afterwards. Or also, if the cataract is very marginal, the benefit that they're getting versus the risk that they're taking might not be right until a bit later on or a few years’ time.”

Participant F went on to say:

“We had quite a lot of anecdotal feedback from people that had been through those services that said, ‘I didn't even know I had a cataract’, but they had it operated on.”

Participant L, located at a different CCG site (setting #3) shared experiences of aggressive eyecare providers exploiting the choice agenda framework. For them, they had experienced the eyecare providers using contracts with much wider specifications (lesser required conditions for treatment) than they would have otherwise agreed to. Central to this theme is the feeling that legally, the commissioners cannot challenge this activity. Whilst trying to improve the health of patients the commissioners are worried that, by challenging risky practices of private providers, they might break competition laws.

“Participant L: But that they have really pushed the choice agenda and the edge of legality… And what they're doing is, they're using specifications from somewhere else in the country where they've got a contract, and then just pulling up and saying, ‘you've got to give us this activity, you've got to put us on your choice menu’… We have to by law, then give them a contract. And they just come to us and say ‘we're doing half a million quids worth of work, you under the regulations have to give us a NHS contract.’”

### 3.2 ‘Leadership appetite’ and personal relationships

Participants regularly expressed the importance of leadership from individuals in the commissioning organisations in whether private sector providers would be used for NHS treatments. This is important because it suggests that a key reason explaining the different levels of outsourcing regionally may be a particularly influential individual in a commissioning body. This is a highly ‘controlled’ form of outsourcing then, as the agency sits very much within the commissioning body – but the motivations are likely to be very personal. Across multiple sites and participants the term ‘appetite’ was used, in similar ways to express a desire to push back against the status quo of NHS provision. It was perceived as a difference of opinion about the best way to meet needs of the population – a kind of philosophy of healthcare provision.

“Interviewer: But I wonder for you, what are the sort of main reasons that you might see a variation [in private sector usage]?

Participant J (CCG setting #2): I don't think you can underestimate individual personalities. So although it might sound like a really silly reason actually, an individual chief exec, or individual chief finance officer, that is quite happy to accept an independent provision in their area could in and of itself make a massive difference”.

Participants from one setting often expressed how their leadership had an appetite which was different to other parts of the country and deeply connected to trying to overcome the poor health outcomes tied to the high levels of deprivation in their area.

“Participant B (CCG setting #1): That was about somebody being brave and innovative. But I think, yeah, in some of the instances, we can do more of the same, or we could, we can really test the market. And I think that [redacted CCG name] from the commissioner's point of view, we've actually been quite strong in saying, ‘let's not shy away from doing something different and taking a risk…

“…something like nine out of 10 of our wards are in the in the bottom decile or whatever for their deprivation nationally, you know that there's a huge amount of poverty. And some money comes with it, but that's what inspired us to do different, be brave, make some decisions that's not usual.”

The stressing of ‘bravery’, ‘innovation’ and being ‘not usual’ in the above quotation, suggests that not all CCGs in highly deprived areas have taken the same approach, but that this was particular to this setting and required a particular type of leadership to bring about.

Beyond the attitudes towards private provision of the individual leaders, several raised working cultures and relationships between the leaders and providers as a possible determinant of outsourcing levels.

“Participant N (CCG setting #3): If you're going to start procuring service, to the private sector, you're going to upset colleagues who you may have gone to medical school with, colleagues with whom you've worked, colleagues with whom you write to on a weekly basis about patients. And so as a leader you've got to have a very thick skin to deal with the criticisms from some colleagues, sometimes from GPs, particularly if they're pals with consultant colleagues and sometimes with other colleagues who feel that this procurement will knock-on to their speciality.”

Sometimes this personal dynamic was described as an attempt to ‘provoke’ the NHS providers. The same participant said:

“[redacted CCG leader] did threaten the hospital with quite a lot of procurement at the very start of [redacted pronoun] tenure, I think that was pushed to shake the hospital up a bit. And to some extent that you can play a bit of tactics, and you can play politics with people, as a leader to try and, well I guess, manipulate essentially…

Sometimes, you know, the hospital were so frustrated with a bunch of colleagues in the hospital themselves, that they quietly said to our senior leadership, why don't you put this out to procurement, that'll really shake this department up”.

### 3.3 Failure of NHS providers in meeting demand: the public sector underfunded and private sector as ‘a release valve’

One perceived reason for outsourcing to the private sector was when the quantity or quality of services were not being achieved in the NHS providers. This type of outsourcing often puts agency with the commissioners and describes situations of pro-active outsourcing – but sometimes sidelines the quality of each provider – suggesting the potential for ‘quality-neglecting’ privatisation. For example, participants reported that if waiting times increased for NHS providers, the private sector would receive more NHS treatments to bring that time back down. Participants M (CCG setting #3) and E (CCG setting #3) explain why it is primarily used to tackle unmet quantity of treatments.

“Participant M: How it works at the moment, more often than not, is it's a release valve, it's a supplier side release valve for demand that has become unmanageable.”

The metaphor of a ‘release valve’ suggests that the power lies with commissioners to ‘switch-on’ and, importantly, ‘switch-off’ private sector usage. It was clear that there are some types of outsourcing and some participants which perceive this process as a primary way the private sector is used, whereas other participants which would stress how their hands were tied by the market and competition regulation.

An important aspect of not being able to meet demand was the long-term underfunding of the NHS. Some commissioners expanded on the ability of private providers to access capital in a way that the NHS could not, meaning that healthcare expansion was often filled by private providers. Participant E (CCG setting #3) said of the private sector:

“If you're an independent sector provider, you can go and look to a venture capitalist company for your start-up monies you can buy or build a property and you can be up and running very, very quickly. Whereas in the NHS, it's a bit of a slow burn. So there is lots of money to be made.”

Another form of capital raised by several participants was the availability of the workforce. Some suggested that workforce was the limiting factor in any attempt to expand public sector provision:

“Interviewer: is there no way to expand public sector provision from the commissioner's point of view?

Participant F (CCG setting #2): So the biggest challenge for us is, is workforce…”

Participant E (CCG setting #3): “we, over the past 12 years have had very restricted access to capital. So new building would be pretty much out of the question. And our ability to grow the workforce, has been, it's not been there really”

Some participants suggested that the demand for high quality – rather than quantity - of services might not be met by the NHS provider. Participant A (CCG setting #3) said: *“Some of it depends on the quality of services. So if you're concerned, or if there's been CQC* [independent health regulator] *issues of serious incidents or complaints”* then *“you are much more likely to go procure for a new service”.* And participant P (CCG setting #3) outlined in an example how poor-quality NHS services could directly result in the outsourcing of a community equipment and wheelchair provision:

“[the services] they were getting were not necessarily as bespoke or as technical as maybe they'd hoped or expected… We can see from some of the data that we've got an issue here, people waiting a lot longer, which results in deterioration in conditions, maybe even shortening of lifespans… And so based on all of those factors, we decided to go out to an open an open tender.”

There were also many times where ‘quality’ and ‘quantity’ were hard to distinguish. It was very common for quality to be measured through the metric of waiting times rather than patient feedback or patient outcomes.

“Participant N (CCG setting #3): where quality tables were poor. Again, NHS managers would be saying, ‘What are you doing about this? The big shiny hospital isn't doing the service well enough, you need to challenge them. Put it out to another provider to get your waiting times down.’”

### 3.4 The austerity paradox: ‘we have no money, we can't outsource’ vs ‘we have no money we have to outsource’

Participants disagreed about the likely overall relationship between levels of CCG funding and the propensity to use the private sector. Some considered it necessary for financially constrained CCGs to procure from the private sector to save money.

“Participant A (CCG setting #3): I think because if you're a financially challenged CCG, which [redacted CCG name] has been for a long time. We've used procurement to try and secure the best value and potentially make savings so potentially we'll have a budget. top slice it a little bit and say that's how much is available. Is there a market out there to provide that service?”

Whereas other commissioners reported that additional resources were required for increased use of the private sector:

“Participant E (CCG setting #3): [During 2000′s Labour government] We had so much money that we couldn't spend it all… And we could commission as much independent sector capacity and NHS capacity as we wanted to achieve that [shorter waiting time targets] because we had the money.”

However, it was clear from the participants that these were very different processes in private sector procurement. Under strict budgets, it was mostly major procurement processes, often for core services, that were put out for competitive tender. Participants also reported that these procurement processes often meant that the NHS provider would win the contract they already held but at a reduced rate.

“Participant N (CCG setting #3): I think it depends on the commissioners financial position, and whether they had enough money to run services well. When you're in financial deficit, and you're struggling to balance the books, the NHS bosses above the, commissioning chiefs, they are always on to saying, ‘hey, gotta save money, you need to cut this service, to put this out to the private sector’.”

Whereas for the commissioners who perceived the opposite relationship (that additional funding enabled private sector outsourcing), the process was about finding providers who could provide ‘enhanced services’ to deliver better and more accessible health services or about increasing the capacity when additional resources are available.

“Interviewer: Just so I understand what you're saying: if you had a CCG that was really financially constrained, they would procure fewer, like add-on, the sort of peripheral services, which are more likely to be provided by the independent sector?

Participant C (CCG setting #3): Yeah, yeah. Yes, because those are the ones that you have the flexibility on, if you see what I mean, although they might be the most important… You might just say, I can't do that anymore. You know, in general practice, we see that we see some enhanced services used to go just get cut year by year… the likelihood is that the most important things [being cut].”

To understand why fiscal constraints might lead to varied private sector usage, I would ask whether private companies offered commissioners cheaper services on average. Commissioners would often respond by saying it was dependent on certain contexts, services, or procurement procedures whether there were savings to be found in the private sector. Participant A (CCG setting #3) said *“I don't think it is something you can generalise”* sometimes if the private provider can act differently such as having online appointments rather than face-to-face, then *“they would inevitably, be cheaper and more flexible”.* But there were many times where competitive tenders would be won by NHS providers because they offered the cheapest services because *“They've already got the network; they've already built up those contractual relationships”* and they had heard of examples when a private provider was used but *“they just didn't last long. You know, they underestimated how much it would cost.”* Similarly, participant D (CCG setting #3) said:

“I would say it's not a generalisation, it may appear cheaper, but it's because they take less complex patients, which are cheaper anyway, whether you're in their full NHS service or not. So, I think it being cheaper is more of an illusion.”

## 4 Discussion

The results of this paper suggest private sector outsourcing can, at times, be grounded in a motivation to improve quality, but that it can also occur as uncontrolled and quality-neglecting outsourcing. In this discussion I will explore which themes provided by the commissioners might represent each scenario.

There were two processes which might prompt concerning forms of privatisation: ’uncontrolled’ and ‘quality-neglecting’ outsourcing. Uncontrolled outsourcing was clearly described by the commissioners when the location and imposition of private providers meant that they could not control the process of healthcare delivery. The example given of ophthalmology providers pushing the choice agenda regulations to the limit when delivering cataracts operations highlighted a powerful way that privatisation was uncontrolled by commissioning bodies. It also highlighted a key moment of ‘quality-neglect’ at a different level - for patients, who, it was suggested, might have been exploited to drive demand and were sometimes given rushed or ill-advised treatments. Commissioners were not always actively selecting the best quality providers because sometimes it was out of their control. This uncontrolled outsourcing may be one way in which privatisation is not improving quality of care. Table 1 categorises each theme based on whether they could represent ‘uncontrolled privatisation’ or ‘quality-neglecting privatisation’ given the location of decision-making power and grounding in healthcare quality.

Many of the results in this paper reflect a period where the health service is underfunded and starved of the required resources by central government – the processes affected by underfunding and austerity were most likely to be a ‘quality-neglecting’ form of privatisation. For instance, outsourcing to reduce waiting times or costs could represent a ‘quality-neglect’ if underfunding has not enabled the best quality provider to deliver the services. The NHS budget has been increasing at a below average rates since 2009 and commissioners have been provided budgets that under evaluate the expected increases in need [34,35]. Underfunding could be underpinning some of the most concerning types of NHS privatisation in England.

### 4.1 Limitations

This paper uses an analysis of commissioner perceptions and experiences to answer why the NHS increasingly uses private providers to deliver treatments. The paper focuses on commissioners because they sit in a position to oversee the mixed market across a region of England, but this focus will inevitably miss experiences of people working in providers, central policymakers, and patients. These other actors may have other insights and experiences leading to alternative explanations of why the NHS uses private providers. A further methodological limitation lies in the solo-coding of the interview data, however peer feedback was received from presentations of the findings in seminars and conferences.

This paper also generates evidence based on 3 key commissioning sites. The findings should be tested for generalisability across the entirety of the NHS in England in future research using quantitative methods. A similar limitation lies in quantifying the extent of each theme and reason for increased privatisation. It was not the aim of this paper to evaluate how much of the increases in privatisation are due to e.g., inadequate levels of NHS funding, compared to the driving of demand by aggressive private providers. Again, future research should use quantitative analysis to explore what the largest reasons are for increases in privatisation.

## 5 Conclusion

The NHS in England is seeing increased levels of privatisation as private providers deliver more treatments year on year. The reasons for this phenomenon in the short-term can often be linked to a deteriorating ability, during an extended period of underfunding, for NHS providers to meet the demands required by the population. In the longer term, marketisation reforms have created a service in which private providers are sometimes not restrained from the NHS and enabled to force the further increases in privatisation.

## Declaration of Competing Interest

The authors declare there is no conflict of interests.

## Appendix Supplementary materials

Image, application 1
