# Supplementary material for: ‘Accident and emergency’? Exploring the reasons for increased privatisation in England's NHS
Source: Health Policy. 2023 Dec;138:104941. doi: 10.1016/j.healthpol.2023.104941 (PMC10933725; doi:10.1016/j.healthpol.2023.104941)
Supplement: Supplementary file 1 [file mmc1.docx]

# APPENDIX A: Participant information and Coding schemes

**Table A1: participant site locations**

| Commissioning site Number (CCG/ICB) | Outsourcing levels | Participant numbers |
| --- | --- | --- |
| 1 | High | H; R; B; Q; O; K; D |
| 2 | High | F; I; J; T |
| 3 | Low | M; P; E; L; G; N; S; C; A |

**Table A2: participant job roles**

| Job Role | Participant numbers |
| --- | --- |
| Leadership team (COO, CFO, deputies, exec committee members) | C; I; F; K; Q; B; P; M; H |
| Lay member | S; G |
| Procurement/ contracting teams | T; A; D; E |
| Commissioning Support | J |
| Clinical/ specialty teams | N; O; L |
| Communications and engagement | R |

**Table A3: basic codes and organising themes**

| Qualitative Analysis | | |
| --- | --- | --- |
| Organising Theme | Basic Theme | Example quotes |
| Failure of NHS providers in meeting demand: the public sector underfunded and private sector as ‘a release valve’ | Longer waiting times increase use of private sector. | “So to produce capacity quickly, to get to 18 week waiting time targets. That was the inception of the independent sector, but that still holds true” |
|  | Varying population demographics would alter need for, and uptake of, private sector. | “But the population [of redacted affluent area] tended to be much more white middle class so there is much more aspirational, of being able to access the private sector, not needing to rely on the NHS... But there is just that aspiration, which the [redacted deprived place name] population don't have the [redacted deprived place name] population are just so pleased that getting anything, they don't have that level of aspiration, whereas [redacted affluent place name] do with it. It's more that middle class image that you're going private” |
|  | Quality metrics and user feedback may lead to use of competitive procurement process. | ““Some of it depends on the quality of services. So if you're concerned, or if there's been CQC issues of serious incidents or complaints, and things like that about a service, then you are much more likely to go procure for a new service.” |
|  | Absence of NHS provision, often for siloed services. | “ if you're an independent sector provider, you can go and look to a venture capitalist company for your start-up monies you can buy or build a property and you can be up and running very, very quickly. Whereas in the NHS, it's a bit of a slow burn.” |
|  | Workforce availability limited NHS expansion. | “I don't think it's realistic for us to think, well, that's okay. We'll just employ 10 other orthopaedic surgeons, because you know, some of what attracts people to work for you in the NHS is the fact that they know that they'll be able to get a certain level of income from the private independent sector market as well.” |
| Private provider locations and the choice agenda | Privatisation driven by locations of private hospitals. | “I think probably the biggest reason is location. And the number of providers around you. You know, it is a hell of a lot easier. Service out for procurement when you've got a massive population and a lot of different providers.” |
|  | New Labour reforms created the situ of private hospitals through ISTC contracts. | “Crikey. It must have been 10 or 15 years ago, the NHS undertook a policy of creating what it described as Independent Treatment Centres, I don't know whether you know about that, for some conditions, so orthopaedics was an obvious one and there was a national procurement exercise which drew in international capacity and capability.” |
|  | Rural commissioners have limited access to private provision. | “trying to attract an independent providers, because we are in such geographical corner, is quite challenging. It is relatively unique to [redacted commissioning place name] because there's very few major cities that are so far away.” |
|  | ‘The Choice Agenda’ and predatory providers empower private providers. | “ So with the choice policy, and Any Qualified Provider policy that been in place in the NHS, then if an Independent Sector provider is contracted by anyone in the English NHS, then they are basically able to set themselves up wherever they want to in the country as an Any Qualified Provider and start basically receiving referrals from your population and you're not able to do anything to stop that” |
| Commissioning Leadership and Politics | Leader’s appetite for alternatives. | “ But the personality of the Chief Exec and chief finance officer, I think can make a massive difference. in a couple of CCGs, a key first question that what we should do is what does the chief exec want to happen? And then, in others it's not, it's, you know, what is what do we think should happen? So, there are some, some very, very strong individual, although it's not formal authority, because authority still will go through the government body voting session, if you had to, actually, the key determining factor in what in what happens is what the chief exec wants to happen.” |
|  | Outsourcing to challenge NHS provider cultures. | “*[redacted CCG leader] did threaten the hospital with quite a lot of procurement at the very start of [redacted pronoun] tenure, I think that will pushed to shake the hospital up a bit. And to some extent that you can play a bit of tactics, and you can play politics with people, as a leader to try and, well I guess, manipulate essentially.”* |
| Consequences of Financing and Austerity | Stringent budgets induce outsourcing | “*When you're in financial deficit, and you're struggling to balance the books, the NHS bosses above the, commissioning chiefs, they are always on to saying, ‘hey, gotta save money, you need to cut this service, to put this out to the private sector’, so on.”* |
|  | Stringent budgets constrain outsourcing | “*The add-on services that you might have looked at in a basket of private procurement. And that could include some enhanced services that came through into general practice. You might just say, I can't do that anymore. You know, in general practice, we see that we see some enhanced services used to go just get cut year by year… the likelihood is that the most important things [being cut].”* |
|  | Prices of comparable services don’t generalise by sector of provision | “*I would say it's not a generalisation, it may appear cheaper, but it's because they take less complex patients, which are cheaper anyway, whether you're in their full NHS service or not. So, I think it being cheaper is more of an illusion.”* |

**A4 – Reflexivity and researcher positionality**

In this paper, I will refer to the levels of private sector provision of NHS treatments as ‘private sector provision’. This reflects language used in the literature, but interviewees would often refer to ‘independent sector providers’ to talk about the same actors. I began the interview process using the term ‘outsourcing’ but adapted to language more in line with what commissioners used, using terms such as ‘procurement’ or ‘provision’. Both ‘outsourcing’ and ‘private sector’ are being used in this paper to make it the most understandable to a broader audience. I will refer to the process of increased private sector provision as ‘privatisation’. This term is taken from the academic literature describing the process of private sector outsourcing, although the interviewees largely avoided using this term.

Interviews were conducted over video calls and the dynamic was that of an outsider researcher interviewing expert participants. I would try not express personal normative views about levels of private sector involvement in the NHS though participants often expressed normative opinions in varying ways on the topic and I would not challenge them. My own positionality was rarely asked about by participants, although I perceived a pre-interview discussion at each commissioning site as a check that I was open-minded to their experiences. I was specifically asked by one participant as to who was funding my research and I inferred that to be a check of whether I had any corporate interests. At the time of conducting interviews, I was simultaneously publishing a research paper finding [redacted for submission]. The paper was never referenced in interviews but the research did shape my thinking about the potential consequences of this policy direction as I began to think about how outsourcing could produce the results of my previous paper.

**A5 - Ethics**

In line with ethical approval from [organisation and ethical reference redacted for submission] the interviews took place via video call and established written informed consent by explaining the research process, data protection and rights to participants. One participant agreed to partake in the research but requested not to be directly quoted. Interviews were recorded with a voice recorder, transcribed, and transferred into NVivo 12.6 software (QSR International Pty ltd 2020). Transcripts had information of individual names, locations, or company and organisation names redacted to achieve pseudonymity. Drafts of the findings were sent to all contactable participants before publication to allow for any additional redactions.

**A6 – Changes in outsourcing broken down by treatment type.**

**
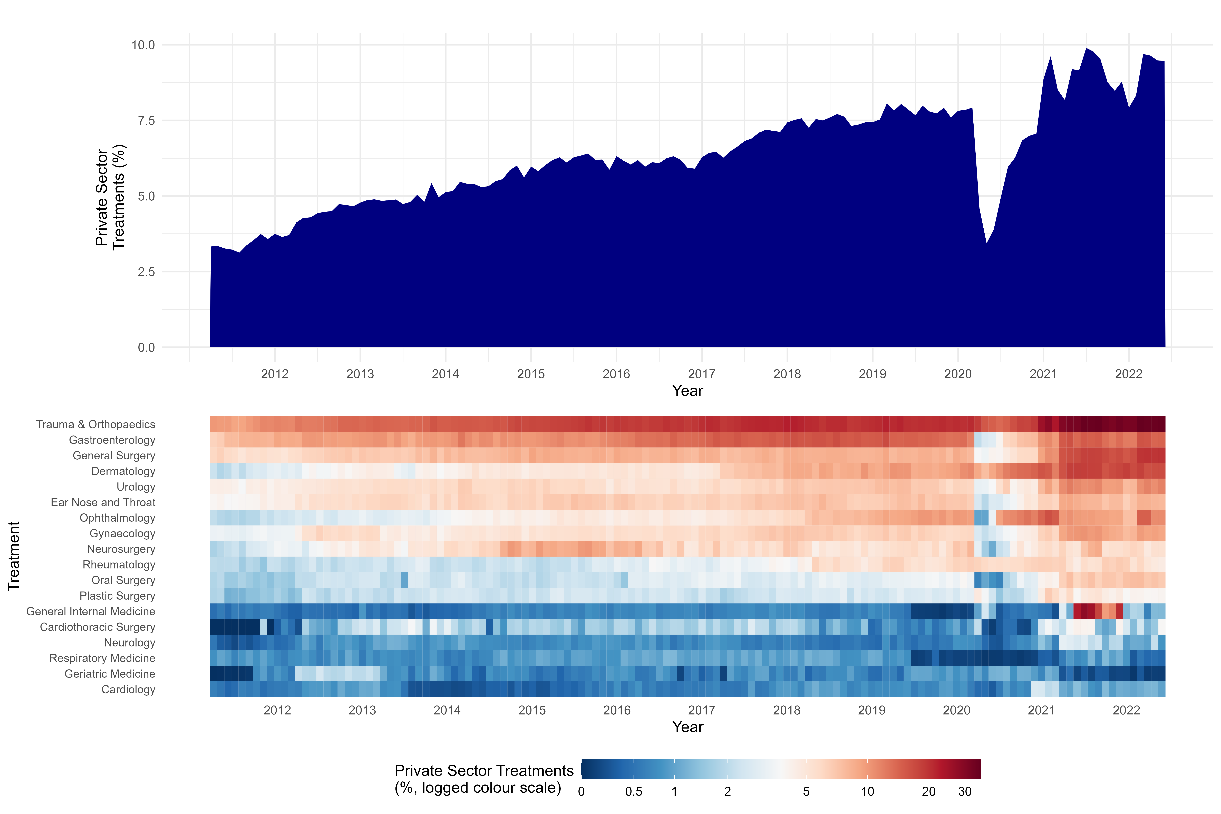
**
